# Bactericidal Activity of Lipid-Shelled Nitric Oxide-Loaded Microbubbles

**DOI:** 10.3389/fphar.2019.01540

**Published:** 2020-01-30

**Authors:** Maxime Lafond, Himanshu Shekhar, Warunya Panmanee, Sydney D. Collins, Arunkumar Palaniappan, Cameron T. McDaniel, Daniel J. Hassett, Christy K. Holland

**Affiliations:** ^1^Department of Internal Medicine, Division of Cardiovascular Health and Disease, University of Cincinnati, Cincinnati, OH, United States; ^2^Department of Molecular Genetics, Biochemistry and Microbiology, University of Cincinnati College of Medicine, Cincinnati, OH, United States; ^3^Department of Biomedical Engineering, University of Cincinnati, Cincinnati, OH, United States

**Keywords:** nitric oxide delivery, lipid-shelled microbubbles, bactericide, USA 300 *Staphylococcus aureus*, bioactive gas delivery, echocontrast agent, ultrasound

## Abstract

The global pandemic of antibiotic resistance is an ever-burgeoning public health challenge, motivating the development of adjunct bactericidal therapies. Nitric oxide (NO) is a potent bioactive gas that induces a variety of therapeutic effects, including bactericidal and biofilm dispersion properties. The short half-life, high reactivity, and rapid diffusivity of NO make therapeutic delivery challenging. The goal of this work was to characterize NO-loaded microbubbles (MB) stabilized with a lipid shell and to assess the feasibility of antibacterial therapy *in vitro*. MB were loaded with either NO alone (NO-MB) or with NO and octafluoropropane (NO-OFP-MB) (9:1 v/v and 1:1 v/v). The size distribution and acoustic attenuation coefficient of NO-MB and NO-OFP-MB were measured. Ultrasound-triggered release of the encapsulated gas payload was demonstrated with 3-MHz pulsed Doppler ultrasound. An amperometric microelectrode sensor was used to measure NO concentration released from the MB and compared to an NO-OFP-saturated solution. The effect of NO delivery on the viability of planktonic (free living) *Staphylococcus aureus* (SA) USA 300, a methicillin-resistant strain, was evaluated in a 96 well-plate format. The co-encapsulation of NO with OFP increased the total volume and attenuation coefficient of MB. The NO-OFP-MB were destroyed with a clinical ultrasound scanner with an output of 2.48 MPa peak negative pressure (*in situ* MI of 1.34) but maintained their echogenicity when exposed to 0.02 MPa peak negative pressure (*in situ* MI of 0.01. The NO dose in NO-MB and NO-OFP-MB was more than 2-fold higher than the NO-OFP-saturated solution. Delivery of NO-OFP-MB increased bactericidal efficacy compared to the NO-OFP-saturated solution or air and OFP-loaded MB. These results suggest that encapsulation of NO with OFP in lipid-shelled MB enhances payload delivery. Furthermore, these studies demonstrate the feasibility and limitations of NO-OFP-MB for antibacterial applications.

## Introduction

Antibiotic resistance is a leading public health challenge of the 21^st^ century (Nolte, 2014; Tacconelli and Pezzani, 2019). Multidrug-resistant bacteria have emerged in both community and nosocomial settings (van Duin and Paterson, 2016), partly due to the misuse of antibiotics in animals and humans as well as horizontal gene transfer (Ventola, 2015). Furthermore, recent efforts toward the development of novel antibiotics have produced diminishing returns (Coates et al., 2011), motivating the need to develop alternative and adjunct strategies for treating bacterial infections. Nitric oxide (NO) is a potent bioactive gas that plays important roles in physiology, including the regulation of vasodilation, platelet activation, and neurotransmission (Elnaggar et al., 2017). NO is downregulated in pathological conditions, such as hypertension, atherosclerosis, and chronic kidney disease (Ahmad et al., 2018). Additionally, NO demonstrates antibacterial activity against a variety of microorganisms (Schairer et al., 2012). Specifically, NO exhibits dose-dependent activity that can not only disperse biofilms but also kill bacteria (Barraud et al., 2009; Schairer et al., 2012; Barraud et al., 2014). Therapeutic exogenous NO delivery has the potential for efficacy against both common and antibiotic-resistant microbial strains *via* reactive intermediates that exert nitrosative and oxidative stresses (Schairer et al., 2012). NO could be used either as a standalone therapy or in combination with other antibacterial agents.

Therapeutic applications of NO are limited by the lack of successful delivery strategies (Elnaggar et al., 2017). NO donors, such as nitroglycerin, are used in the clinic to manage acute hypertension (Flaherty et al., 1982). However, nitroglycerin can cause side effects, such as a systemic reduction in blood pressure, which precludes broad usage (Bloch et al., 2007). Inhalation of NO has been used to treat hypoxemia and pulmonary arterial hypertension in full-term and near-term neonates (Ichinose et al., 2004; Bloch et al., 2007). Additionally, recent studies suggest that NO inhalation can prevent chronic lung disease in premature infants and alleviate ischemia-reperfusion injury (Bloch et al., 2007; Kida and Ichinose, 2014). However, inhalation is expensive, cumbersome and suitable for a limited number of applications (Thunberg et al., 2015). Therefore, the development of site-specific and triggered delivery of NO is an active area of research (Elnaggar et al., 2017). Biomaterials, such as dendrimers (Sun et al., 2012), sol-gels (Robbins and Schoenfisch, 2003), polymers (Studenovsky et al., 2018), and lipids (Elnaggar et al., 2017), are under investigation as carriers for NO donors. Loading of NO in micron-sized microbubbles (MB) is another strategy for delivery of this bioactive gas (Wang et al., 2013; Sutton et al., 2014; Grishenkov et al., 2015). Echogenic liposomes containing MB encapsulated in multilamellar structures or monolayers have also been used for NO delivery (Huang et al., 2009; Kim et al., 2014; Lee et al., 2014).

The theoretical considerations for NO delivery using MB and ultrasound have been explored previously (Postema et al., 2006). Intravenous release of NO from lipid-shelled MB was shown to accelerate the resolution of deep vein thrombosis in a murine model (Wang et al., 2013). NO-loaded MB have been used with stem cells for regenerating ischemic tissue after myocardial infarction (Tong et al., 2013). NO-loaded echogenic liposomes (NO-ELIP) have been tested for the treatment of breast cancer *in vitro* (Lee et al., 2014). NO-ELIP were also explored to trigger vasodilation (Kim et al., 2014), and to reduce neointimal hyperplasia (Huang et al., 2009). In a review on ultrasound-mediated therapeutic delivery for the treatment of biofilm in chronic wounds, LuTheryn et al. (2019a) describe the potency of NO for biofilm dispersion and bactericide applications, and highlight the need for site specific delivery of NO. LuTheryn et al. suggest sonosensitive NO-loaded MB as a strategy for controlled delivery. The same group notably reported preliminary data on biofilm dispersion using NOMB (LuTheryn et al., 2019b).

Recent studies on bioactive gas delivery suggest that the bioactive gas payload can be stabilized by the co-encapsulation of perfluorocarbon gases (Kwan et al., 2012; Yang et al., 2018; Shekhar et al., 2019b). Whether co-encapsulation of a perfluorocarbon gas can stabilize NO payload within MB has not been determined previously.

In this study, we evaluated the amount of NO loading, acoustic response, and stability of MB synthesized with either NO alone (NO-MB), or with NO and OFP at different volume fractions: 90% NO and 10% OFP (NO-OFP-MB 9:1 v/v), or 50% NO and 50% OFP (NO-OFP-MB 1:1 v/v). The size distribution and acoustic attenuation of NO-loaded MB were characterized. The NO dose delivered using these agents also was measured. Next, the imaging and release of the gaseous payload of MB was demonstrated using a commercial ultrasound scanner, exhibiting compatibility of this technique in a clinical setting. Finally, we evaluated the feasibility of killing the USA 300 strain of *Staphylococcus aureus* (*S. aureus*) using NO-loaded MB *in vitro*.

## Materials and Methods

### Preparation of Lipid-Shelled MB

All lipids were purchased from Avanti Polar Lipids (Alabaster, AL, USA). NO (99.99%) and OFP (99.9%) gases were sourced from Wright Brothers (Ohio, USA) and Advanced Specialty Gases (Reno, NV, USA), respectively. Bovine serum albumin (BSA), propylene glycol, and glycerol were acquired from Sigma Aldrich (St. Louis, MO, USA). To prepare NO-loaded MB, 1,2-distearoyl-sn-glycero-3-phosphocholine (DSPC), and 1,2-distearoyl-sn-glycero-3-phosphoethanolamine-N-[methoxy(polyethylene glycol)-2000] (18:0 PEG2000 PE) were combined in a molar ratio of 9:1. The procedure for preparing these lipid-shelled MB has been reported in detail previously (Shekhar et al., 2019b). Briefly, a thin lipid film was formed in a round bottom flask using a rotary evaporator (N-1001, Eyela, Bohemia, NY). Thereafter, the residual solvents were removed by overnight lyophilization (Labconco FreeZone 2.5, Labconco, Kansas City, MO, USA). Subsequently, the lipids were rehydrated using a solution of PBS:propylene glycol:glycerol at 16:3:1 volume ratio that was pre-warmed to 60°C to obtain a lipid concentration of 1 mg/ml. After rehydration, the lipid suspension was sonicated (Branson 3510, Branson Ultrasonics, Danbury, CT, USA) for 30 min to obtain a lipid dispersion. Aliquots (1.4 ml) of this dispersion were transferred into serum glass vials (3 ml total volume, item# 223683, Wheaton, Millville, NJ USA), sealed using gas-tight butyl-rubber stoppers, and crimped. Similarly, 1.4-ml aliquots of the buffer solution were stored in 3-ml serum vials, sealed, and crimped. The air in the headspace of each vial was evacuated for 30 s to a pressure of 40 mmHg using a vacuum pump (model number: 8803, Welch Vacuum Technologies Inc., Mt. Prospect, IL, USA). Subsequently, the vials were refrigerated at 4°C and used for experiments within 2 weeks.

Vials were warmed to room temperature for 1 h before adding the gases. NO and OFP gases were collected in 0.5-L gas-sampling bags (Tedlar^®^, Zefon International, Ocala, FL, USA). The 1.6-ml vial headspace was filled with either NO only, or a mixture of NO and OFP (9:1 and 1:1 v/v), or a mixture of air and OFP 9:1 v/v using a 3-ml syringe fitted with a 30-G needle. Thereafter, the vials were activated by high-shear mixing for 45 s with a VIALMIX™ device (Lantheus Medical Imaging, N. Billerica, MA, USA) to produce NO-MB and NO-OFP-MB. As the high-shear mixing process generated heat, the vials were allowed to cool for 15 min to room temperature before use.

### Size Distribution Assessment

A Coulter counter (Multisizer 4, Beckman Coulter, Brea, CA, USA), equipped with a 30-µm aperture, was used to measure the size distributions of NO-MB and NO-OFP-MB, as described previously (Shekhar et al., 2018) at room temperature. NO-MB or NO-OFP-MB were diluted 1,000 and 20,000-fold in air-saturated PBS, respectively. The size distributions of three vials of each type of microbubble were assessed (n = 3). The volume-weighted size distribution histograms were obtained and adjusted for the degree of dilution. The total volume of gas in the MB was calculated from the volume-weighted size distribution measurements.

### Differential Interference Contrast Microscopy

Differential interference contrast (DIC) microscopy was performed to assess the morphology of lipid-shelled MB. Either NO-MB, or NO-OFP-MB at 1:1 or 9:1 v/v NO-OFP ratio (10 μl, undiluted) were added to a polystyrene (plasma-treated) microscope slide (Ted Pella, Redding, CA, USA), covered with a glass coverslip, and visualized with DIC microscopy using an Axioplan two imaging microscope (Zeiss, Thornwood, NY, USA). A 63× oil-immersion objective (Plan Apochromat, Zeiss, Thornwood, NY, USA) with a numerical aperture of 1.4 was used along with a charged coupled device camera (Axiocam MRM, Zeiss, Thornwood, NY, USA) to acquire images. Images of Definity^®^, an FDA-approved echo-contrast agent, were also acquired under the same conditions for comparison.

### Acoustic Attenuation and Ultrasound-Triggered Release

**Figure 1:**
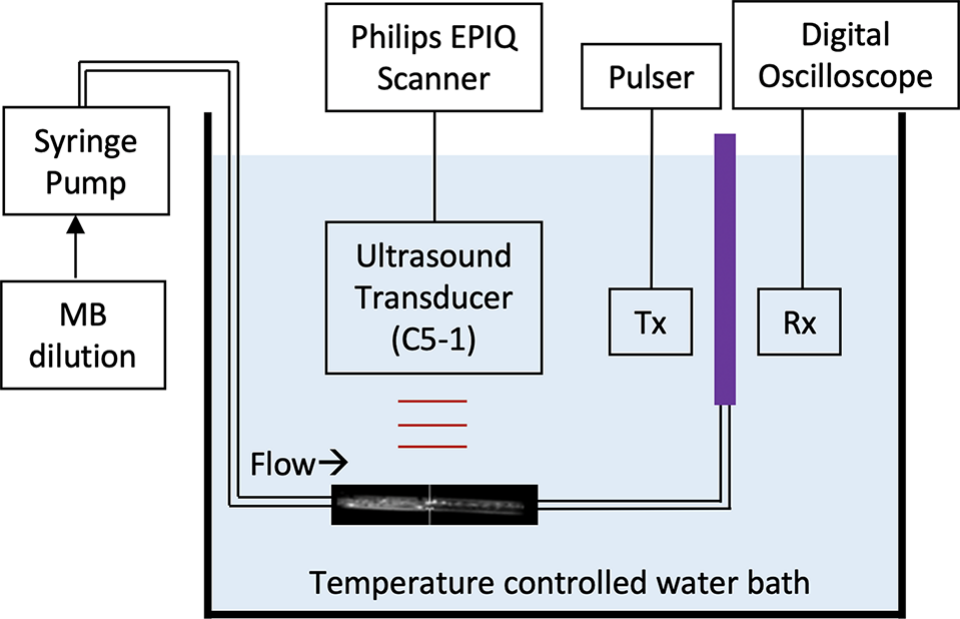
Schematic of the system used for broadband acoustic attenuation spectroscopy. The purple region denotes the CLINIcell sample chamber. Tx and Rx denotes the transmitting and receiving transducers, respectively.

Broadband attenuation spectroscopy was used to measure the acoustic attenuation coefficient of NO-MB and NO-OFP-MB from 2 to 25 MHz at 37 °C as reported previously (Raymond et al., 2014). The attenuation spectroscopy system is shown in **Figure 1**. Briefly, the agents were diluted 2,000-fold in a 37 ± 0.5 °C solution of 0.5% BSA in air-saturated PBS. The through-transmission attenuation spectrum was measured using a broadband transducer pair (PI-20, Olympus NDT, Waltham, MA, USA). The same system was used along with two ultrasound insonation schemes to assess the ultrasound-triggered release of the gas payload from NO-OFP-MB. Specifically, the NO-OFP-MB (9:1 or 1:1 v/v) diluted in BSA were perfused through a 1-mm inner diameter ethyl vinyl acetate tube (McMaster-Carr, Aurora, OH, USA) at a rate of 11.7 ml/min, filling the cell-culture cassette serving as a measurement chamber (CLINIcell 25, Mabio, Tourcoing, France) in approximately 30 s. A C5-1 transducer array (Phillips, Bothell, MA, USA) with a center frequency of 3.41 MHz attached to a clinical ultrasound scanner (EPIQ 7G, Philips Healthcare, Bothell, WA USA) was positioned 1 cm away from the tube. The acoustic attenuation of NO-OFP-MB was measured after exposure to either sham (no ultrasound), B-mode ultrasound at an on-screen MI of 0.04 (Porter et al., 2006; Radhakrishnan et al., 2012), or Duplex Doppler ultrasound at an on-screen MI of 1.2. Exposure or sham exposure was performed in the tube between the syringe and the measurement chamber shown in **Figure 1**. The peak negative pressure and *in situ* MI within the tube were measured using a 0.2 µm needle hydrophone (Precision Acoustics, Dorchester, UK). The NO-OFP-MB were exposed to B-mode pulses at a peak negative pressure of 0.02 MPa (or an *in situ* MI of 0.01), or Duplex Doppler ultrasound at a peak negative pressure of 2.48 MPa (or *in situ* MI of 1.34). Complete attenuation loss was used to indicate gas payload release from lipid-shelled MB. All the attenuation measurements were performed in triplicate.

### Quantification of NO Loading

**Figure 2:**
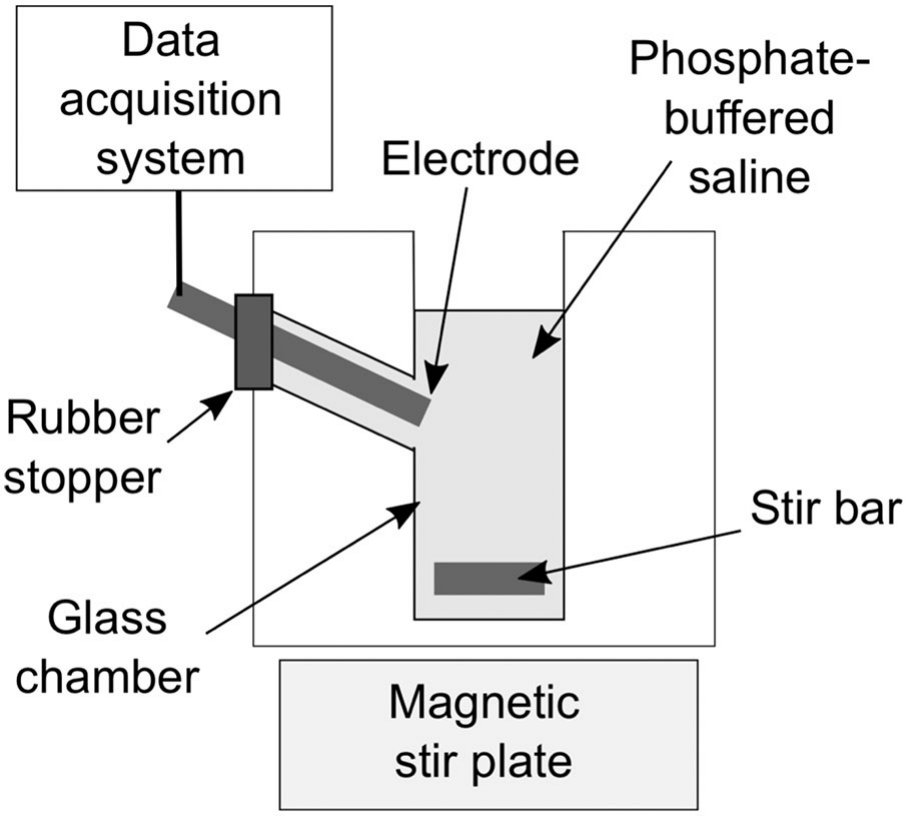
Schematic of the experimental set-up with the amperometric microelectrode sensor that was used to measure the NO dose.

The NO dose in the MB was quantified using an amperometric microelectrode sensor (Apollo 4000 with ISO-NOP electrode; World Precision Instruments, Sarasota, FL, USA), which had a response time of about 5 to 7 s, using the experimental set-up shown in **Figure 2** at room temperature. NO and OFP gases were removed from compressed lecture bottle-sized cylinders and transferred to gas sampling bags. Standard solutions with known gas volume fractions (v/v) were created by diluting NO-saturated water in PBS. The concentration of NO in NO-saturated water was calculated based on the published solubility coefficient at room temperature, 1.91 mM. The peak electrode response was evaluated for a range of NO-saturated solution volumes between 2 µl and 20 µl. A linear regression was performed to obtain a calibration curve relating the concentration of NO with the measured peak response of the amperometric microelectrode sensor.

**Figure 3:**
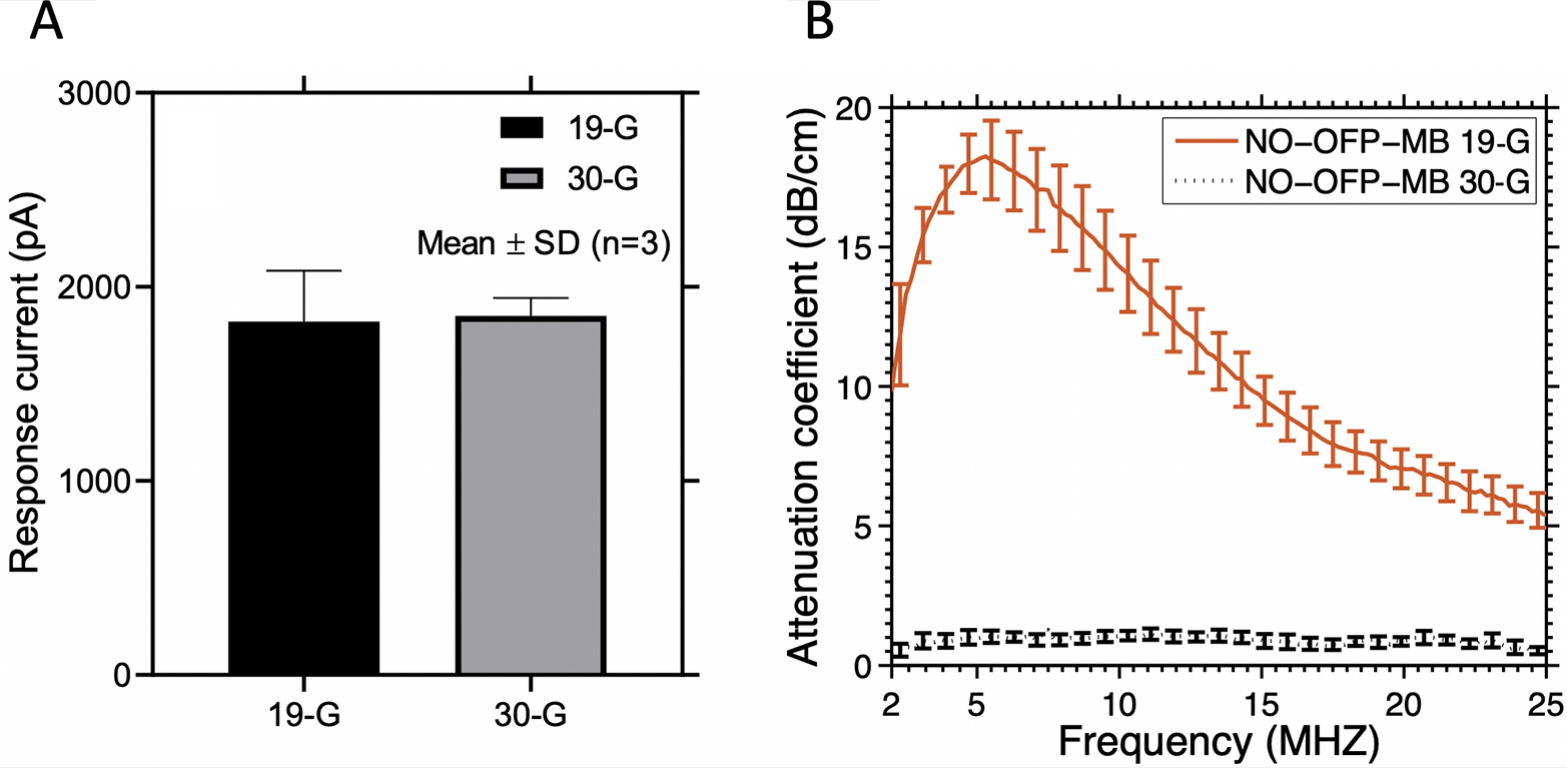
**(A)** The response current of the amperometric microelectrode sensor to NO-saturated solution obtained after injection through a 19-G or 30-G needle. **(B)** Attenuation coefficient of NO-OFP-MB 9:1 v/v obtained after injection through a 19-G or a 30-G needle. Injection through a 30-G needle resulted in a sharp decrease in the attenuation coefficient, and release of the gas payload. The mean ± 1 standard deviation is depicted (n = 3 per group) in each case.

To determine the NO dose in NO-OFP-MB, 10 µl of the dispersion were added to 2.5 ml of PBS in a glass measurement chamber, with and without destroying the MB. A protocol based on injection of NO-OFP-MB through a 30-G needle into the glass measurement chamber was used to destroy the MB and release the gas payload. A preliminary experiment was performed to validate this injection protocol (**Figure 3**). A 10-µl aliquot of NO-saturated solution was drawn into a glass syringe through a 19-G needle and injected into the measurement chamber through the same needle. Next, a 19-G needle was used to draw the solution and the syringe was replaced with a 30-G needle, ensuring that a 15-µl solution was present in the syringe before injection. This procedure allowed us to account for the dead volume difference between the 30-G and the 19-G needles (5 µl). Thus, 10-µl solution was delivered into the measurement chamber. Both injection techniques resulted in the same current response from the amperometric microelectrode sensor (*p* > 0.05, **Figure 3A**, n = 3). Next, it was determined whether injection through a 30-G needle destroyed the MB (**Figure 3B**). The attenuation coefficient of NO-OFP-MB 9:1 v/v was determined after injection using a 19-G and a 30-G needle into BSA (1:3000 dilution, n = 3). These measurements showed almost complete loss of attenuation of NO-OFP-MB 9:1 v/v after injection through a 30-G needle (**Figure 3B**).

Subsequent to the validation of the injection protocol, the NO dose in NO-OFP-MB (9:1 v/v and 1:1 v/v) was measured using the amperometric microelectrode sensor (n = 9 except for NO-OFP-MB 9:1 v/v with 30-G needle: n = 10). The concentration of NO measured by the amperometric microelectrode sensor was corrected for the dilution factor and reported in terms of in vial concentration in mM. As an alternate measurement method, the NO concentration was also determined from the size measurements obtained using the Coulter counter. The size measurement was performed after diluting the bubbles in an air-saturated PBS solution, which initiated gas diffusion into and out of the MB across the lipid shell with a concomitant change of microbubble size. Hence, NO-OFP-MB size and gas content dynamics of the bubbles injected in the PBS for measurement were modeled using a multi-gas bubble model taking into account the NO-OFP loading in an air-saturated fluid (oxygen plus nitrogen) (Kwan et al., 2012). Briefly, this model describes gas exchange through the bubble layers (1) and the expression for the mechanical equilibrium (2):

(1) $$\begin{array}{c} \frac{dn_{i}}{dt}=-\frac{4\pi R^{2}K_{H,i}}{\Omega_{s,i}+\Omega_{w,i}+R_{PEG}}\left(P_{H}+P_{L}-\frac{3BT}{4\pi R^{3}}\sum_{j}^{N}n_{j}-f_{i}P_{sat}\right) \end{array}$$

(2) $$\begin{array}{c} \frac{3BT}{4\pi R^{3}}\sum_{i=1}^{N}n_{i}=\frac{2\sigma }{R}+P_{H}, \end{array}$$

where *n* is the moles of gas inside the bubble, *R* is the bubble radius, *K_H_* is the Henry's constant of each gas (denoted by the subscript *i*). *P_H_* is the hydrostatic pressure (101325 Pa), *P_sat_* is the saturation pressure, *P_L_*=2*σ*/*R* is the Laplace pressure with *σ* the surface tension, and *f* is the gas saturation fraction in the medium surrounding the bubble. *B* is the ideal gas constant, and *T* is the temperature. *N* is the number of gas species considered. The subscript *j* denotes a different gas species (*j*≠*i*). Ω*_s_*, and *R_PEG_* denote resistance to gas diffusion from the lipid shell, and the pegylation (Borden and Longo, 2002), respectively. Ω*_w,i_*=*R*/*D_i_* is the resistance of gas diffusion in the bulk medium, and *D* is the diffusivity of each gas species, *i*, in the bulk medium. The resistance to diffusion (in s/cm), Ω*_s,i_*, was implemented in an energy barrier model by Kwan et al. (Kwan et al., 2012):

(3) $$\begin{array}{c} \Omega_{s,i}=\Omega_{n}e^{\frac{\pi a_{i}^{2}}{k_{B}T}\left(\sigma_{0}-\sigma \right),} \end{array}$$

where Ω*_n_*=133*s*/*cm* (Kwan et al., 2012) is a constant associated with the lipid encapsulation, *a_i_* is the collision diameter of the gas molecule, *k_B_* is the Boltzman constant, and *σ*_0_=0.073*N*/*m* is the surface tension of the gas-liquid interface. Custom code was written in Matlab^®^ (MathWorks), run on a desktop personal computer, and used to solve equations (1) and (2) iteratively. Equation (1) was solved using a finite difference scheme. The polynomial equation (2) was solved by finding the eigenvalues of the companion matrix in rational canonical form using the Matlab^®^ function “root” and selecting the only real and positive solution. Initial bubble radii between 0.5 and 30 µm were modeled. The gas properties used in the computation are listed in **Table 1**. We assumed that the MB reached minimum sizes before measurement with the Multisizer 4 (Pu et al., 2006). Thus, the measured bubble size distribution was corrected for this size change due to gas diffusion and used to calculate the expected NO dose. The kinetics of the reaction of NO with oxygen in aqueous solution was modeled using the following set of equations adapted from Lewis and Deen (1994):

(4) $$\begin{array}{c} \frac{d\left[NO\right]}{dt}=-4k_{1}{\left[NO\right]}^{2}\left[O_{2}\right]+{\left(\frac{k_{G}A_{G}}{V}\right)}_{NO}\left({\left[NO\right]}^{*}-\left[NO\right]\right)\;,\; \end{array}$$

(5) $$\begin{array}{c} \frac{d\left[O_{2}\right]}{dt}=-k_{1}{\left[NO\right]}^{2}\left[O_{2}\right]+{\left(\frac{k_{G}A_{G}}{V}\right)}_{O_{2}}\left({\left[O_{2}\right]}^{*}-\left[O_{2}\right]\right)\;,\; \end{array}$$

**Table 1:** Gas properties used in the simulations.

Gas properties	Oxygen	Nitrogen	Octafluoropropane	Nitric oxide
Henry's constant - *K_H_* [*mol.m*^-3^.*Pa*^-1^] (×10^-6^), derived from Ostwald coefficients (Wilhelm et al., 1977; Kwan et al., 2012)	7.56	7.14	0.21	19.00
Collision diameter - *a* [*Å*]	3.46 (Ismail et al., 2015)	3.64 (Ismail et al., 2015)	6.95 (Siebert and Knobler, 1971; Sarkar et al., 2009)	3.17 (Ismail et al., 2015)
Diffusivity – *D*[*m*^2^.*s*^-1^] (×10^-9^)	2.10 (Cussler and Cussler, 2009)	1.88 (Cussler and Cussler, 2009)	0.745 (Sarkar et al., 2009)	2.21 (Zacharia and Deen, 2005)

where *k*_1_ is the rate constant of the reaction (2.1×10^6^M^-2^s^-1^), and *k_G_A_G_*/*V* is the volumetric mass-transfer coefficient (2.8×10^-3^s^-1^ and 2.5×10^-3^s^-1^ for NO and oxygen, respectively). The asterisks denote the concentrations in the gas phase. The concentration of NO in the gas phase, [*NO*]^*^, was assumed to be negligible. The expected NO dose was derived from the size distribution measurements (n = 3) and assumed to be the initial NO concentration.

The stability of the “in vial” NO dose was assessed by successive measurements 15 min, 2, 4, and 6 h after vial activation (n = 3). The NO dose in a 96 well-plate was measured between 15 and 340 s (n = 3). The results were compared with the model of gas diffusion including the reaction of NO with oxygen.

### Susceptibility of *Staphylococcus Aureus* Strain USA300 to NO-OFP-MB

The susceptibility of *S. aureus* USA300, a methicillin-resistant strain, to NO-OFP-MB 9:1 v/v was evaluated *in vitro*. Because NO-OFP-MB 9:1 v/v had the highest NO dose determined by the experiments described in section 2.5, this formulation was used for bactericidal assessment. Air-OFP-MB 9:1 v/v served as a control to assess the effect of pegylated lipid-shelled MB without NO on the bacteria. *S. aureus* strain USA300 bacteria were grown on Luria-Bertani (LB) agar and maintained at 37 °C overnight. Thereafter, a single colony of bacteria was used to inoculate Mueller Hinton (MH) broth for 18 h then 100-fold diluted with 20% MH broth in a 96 well-plate. Subsequently, 100 µl of either air-saturated solution (consisting of PBS, propylene glycol, and Glycerol mixed at 16:3:1 v/v ratio), or NO-saturated solution, or air-OFP-MB 9:1 v/v, or NO-OFP-MB 9:1 v/v were added to each well plate to obtain a total volume of 200 μl. Each vial was sealed and vented to a bag of the corresponding gas mixture to avoid degradation of NO by O_2_ between replicates. The plates were incubated 24 h at 37°C, and the viability of the cells was counted using the serial dilution method. A 20 μl aliquot of each sample was transferred into each well of a 96 well-plate. Subsequently, bacteria were diluted 10-fold in PBS, and 10 μl of each diluted sample was spotted on an LB agar plate. After incubation at 37 °C for 18 h, bacteria colonies were counted and reported as colony forming units (CFU)/ml. At least nine independent bactericide experiments were performed using fresh vials and a different colony of bacteria each time over three different days of experiments.

### Statistical Analysis

All statistical analyses were performed on GraphPad Prism version 8.0.2 for MacOS (GraphPad Software, San Diego, CA, USA). The data are reported as the mean ± 1 standard deviation. To determine whether the differences in particle volume and payload of NO in NO-MB or NO-OFP-MB (9:1 v/v or 1:1 v/v) were statistically significant, ANOVA tests with Dunn's multiple comparison correction were employed after testing for normality using a Kolmogorov-Smirnov test. No outliers were detected using the robust regression and outlier removal (ROUT) method with the parameter Q set to 1% (recommended setting in GraphPad Prism). For bacteria killing experiments, the data were tested against a normal distribution using the Kolmogorov-Smirnov normality test and three outliers were removed using the ROUT method. The Kruskal-Wallis test was used to compare the CFU/ml obtained with air-saturated solution, NO-OFP 9:1 v/v saturated solution and air-OFP-MB against NO-OFP-MB. Post-hoc Dunn's multiple comparison test was used to obtain adjusted *p*-values. A *p*-value less than 0.05 was considered statistically significant.

## Results

**Figure 4:**
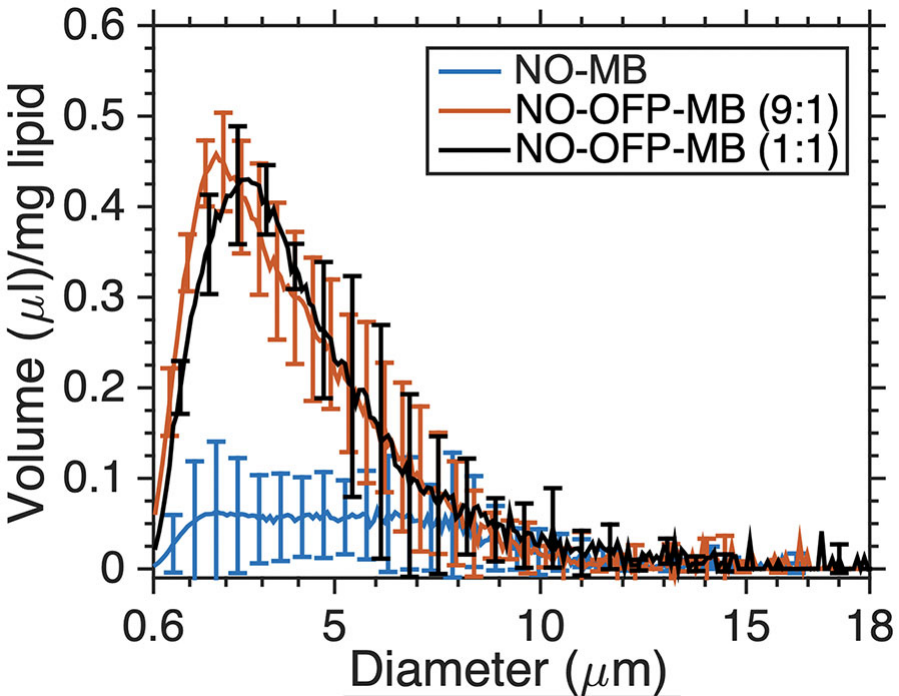
The volume-weighted size distribution of NO-MB and NO-OFP-MB (9:1 v/v and 1:1 v/v). The mean ± 1 standard deviation is depicted (n = 3 per group).

**Figure 4** shows the volume-weighted size distributions of NO-MB and NO-OFP-MB (9:1 and 1:1 v/v). The shape of the volume size distribution of NO-OFP-MB was similar for the 1:1 and 9:1 ratio of NO and OFP. However, the total volume of NO-MB was significantly lower than NO-OFP-MB (*p* < 0.01).

**Figure 5:**
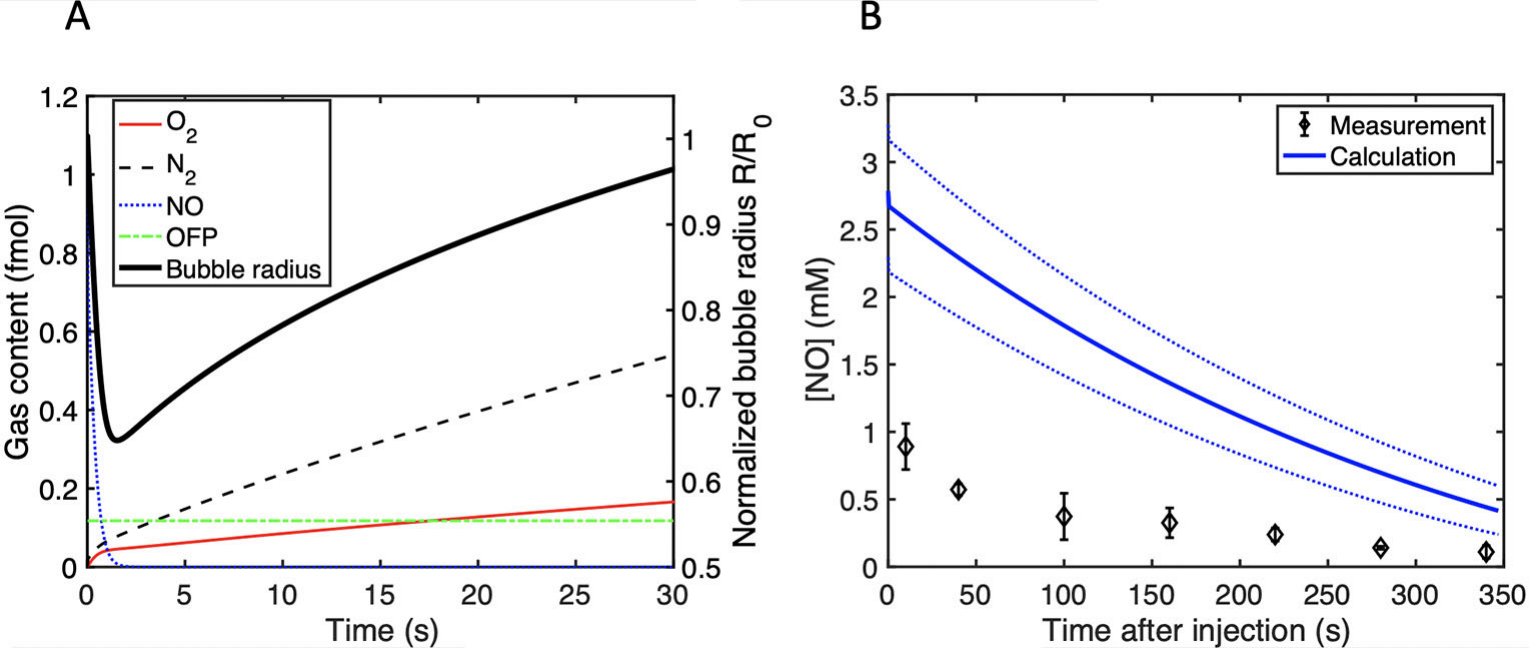
**(A)** The gas content in femtomoles inside a single 3.5-µm diameter bubble and the normalized radius as a function of time after immersion in air-saturated water. **(B)** Calculation of the time-dependent NO concentration in the 96 well-plate used to assess bactericidal activity. The initial NO concentration was determined from the size measurements. The dotted lines represent the range of the NO concentration calculated based on the standard deviation in the size distribution measurements.

Simulations of the gas content and radial changes of a 3.5-µm diameter bubble immersed in air-saturated water are shown in **Figure 5A**. In the first 2 s, NO diffused out of the microbubble, and the radius dropped by 44%. Conversely, oxygen and nitrogen diffused into the microbubble. OFP diffusion is not significant over 30s. **Figure 5B** shows the predicted and measured concentration of NO in a 96-well plate. This predicted drop in NO concentration is higher than the measured values by a factor of approximately 2–3.

**Figure 6:**
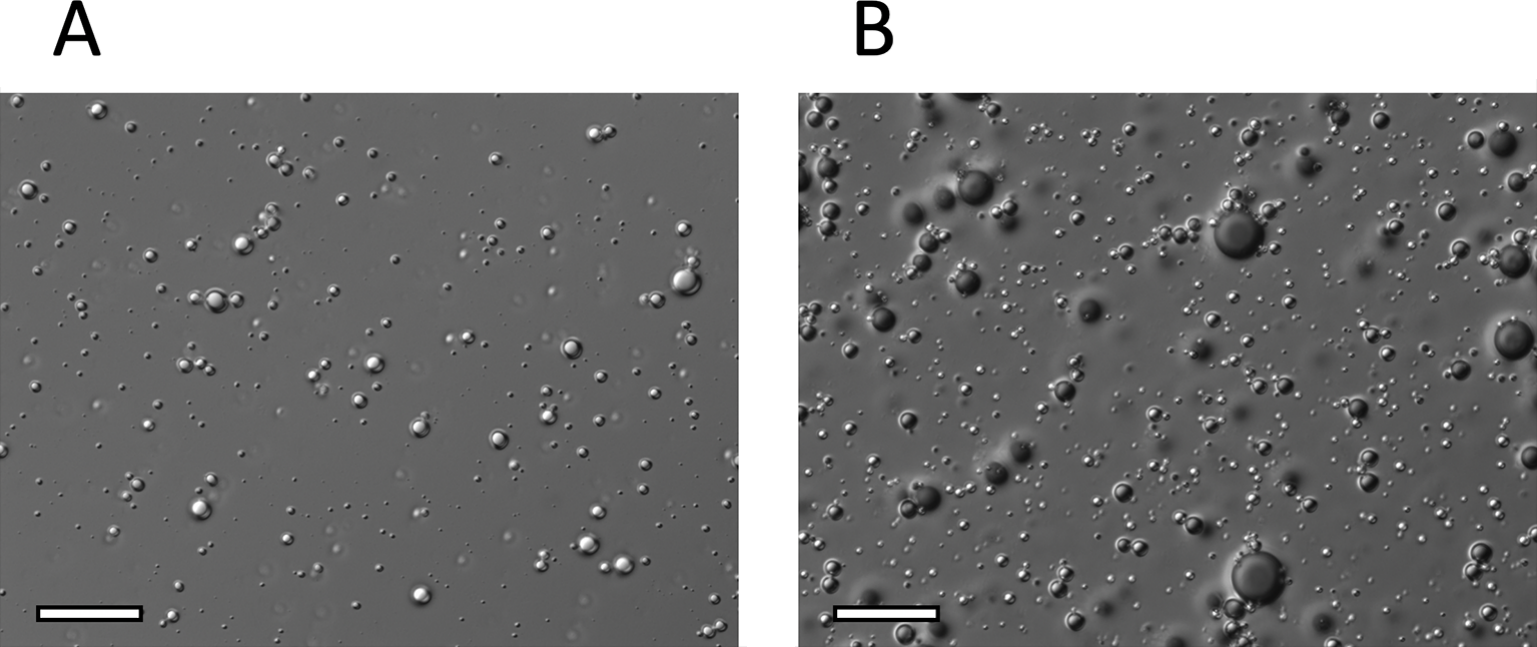
Representative DIC microscopy images (x 63) of **(A)**, NO-OFP-MB 9:1 v/v, and **(B)**, NO-OFP-MB 1:1 v/v. Scale bar = 20 µm. NO-OFP-MB, Nitric oxide and octafluoropropane microbubbles.

DIC images of NO-OFP-MB are shown in **Figure 6**. NO-MB were not visible on the microscope. The NO-OFP-MB 9:1 v/v are both smaller and sparser than the NO-OFP-MB 1:1 v/v. Both agents were polydisperse in size, consistent with the high-shear mixing process of preparation (Borden, 2016).

**Figure 7:**
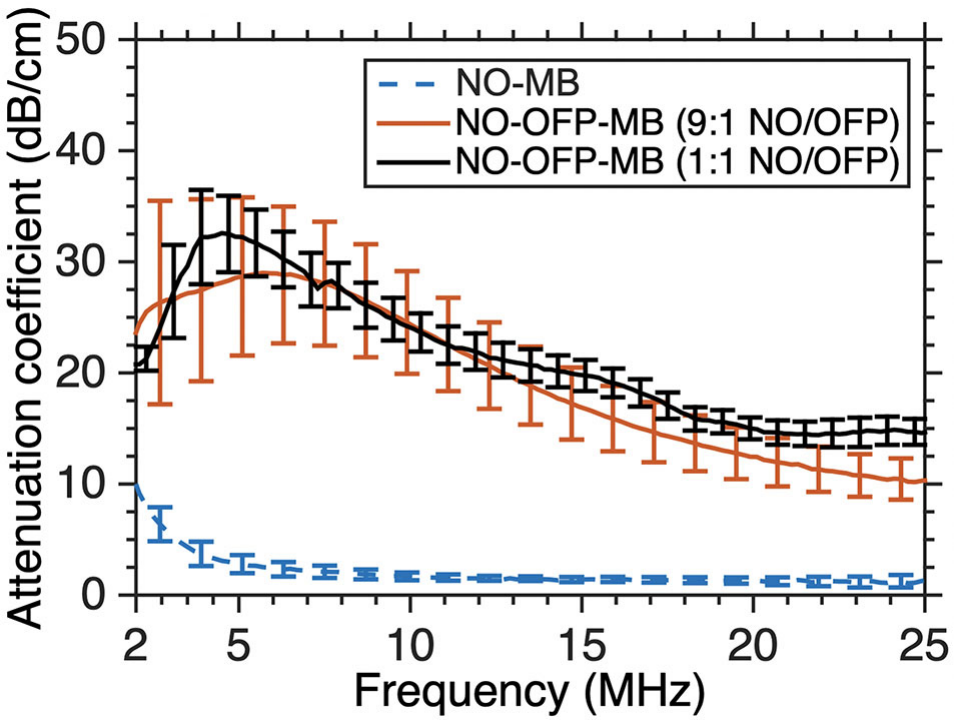
The frequency-dependent attenuation spectra of NO-MB and NO-OFP-MB (9:1 v/v and 1:1 v/v). The mean ± 1 standard deviation is depicted (n = 3 per group). NO-OFP-MB, Nitric oxide and octafluoropropane microbubbles.

**Figure 8:**
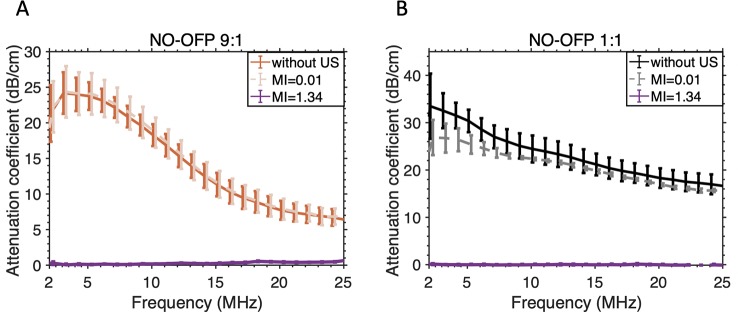
Attenuation-coefficient of NO-OFP-MB [**(A)** 9:1 v/v, **(B)** 1:1v/v] without ultrasound or after exposure to ultrasound at MI=0.01 (used for imaging) or MI=1.34 (ultrasound-triggered gas payload release). The mean ± 1 standard deviation is depicted (n = 3). NO-OFP-MB, Nitric oxide and octafluoropropane microbubbles.

**Figure 7** shows the attenuation coefficient of NO-MB and NO-OFP-MB. The attenuation coefficient of NO-MB was substantially lower than NO-OFP-MB. However, the attenuation coefficient of NO-OFP-MB prepared with the 1:1 and 9:1 v/v ratio of NO and OFP was similar. In **Figure 8** the frequency-dependent attenuation coefficient of NO-OFP-MB 9:1 v/v before and after exposure to B-mode (0.02 MPa peak negative pressure or *in situ* MI of 0.01), and 3-MHz pulsed duplex Doppler ultrasound (2.48 MPa peak negative pressure or *in situ* MI of 1.34), is plotted. The MB retained attenuation after exposure to B-mode ultrasound at an *in situ* MI of 0.01. However, a precipitous loss in attenuation was observed after exposure to pulsed duplex Doppler ultrasound (*in situ* MI of 1.34).

**Figure 9:**
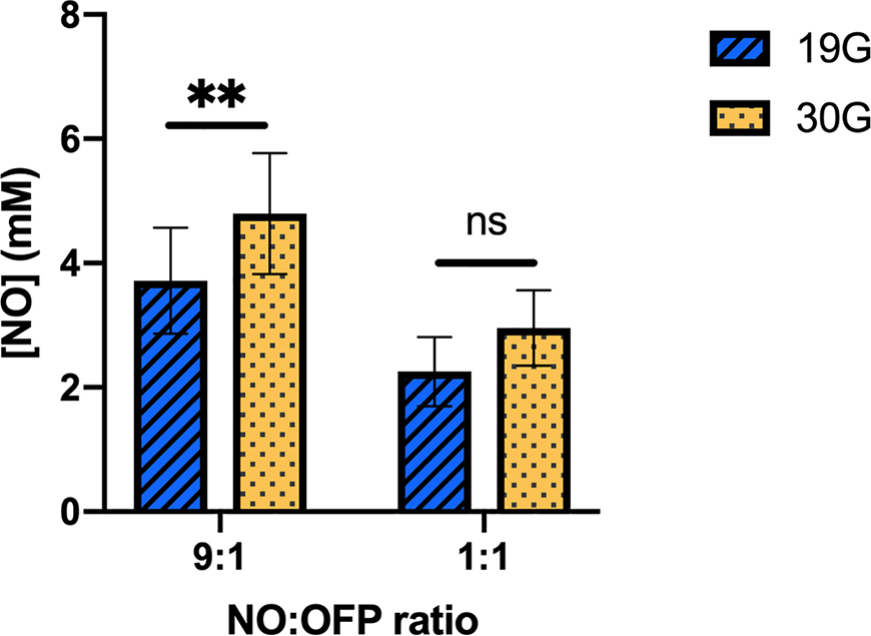
The NO concentration in the NO-OFP-MB (9:1 and 1:1 v/v). The mean ± 1 standard deviation is depicted. n = 9 per group except for 9:1 30-G (n = 10). The symbols ‘ns', and ‘**' represent *p*-values > 0.05, and < 0.01, respectively. NO-OFP-MB, Nitric oxide and octafluoropropane microbubbles. The difference between panel A and B is that A is 9:1 v/v and B is 1:1 v/v. Please change caption to read, "(Panel A: 9:1 v/v, Panel B: 1:1 v/v)".

**Figure 9** shows the measured concentration of NO in NO-OFP-MB (9:1 and 1:1 v/v) per ml of solution. Water saturated with a combination of NO and OFP either at 1:1 or 9:1 v/v mixture ratio contains 0.96 or 1.72 mM NO at room temperature (Manahan, 2017). The NO dose of NO-OFP-MB 1:1 and 9:1 v/v was 3.1 and 2.8-fold higher than that of water saturated with an equivalent NO and OFP mixture ratio. The NO dose in NO-OFP-MB measured after injection with a 30-G needle was higher than the dose after injection with a 19-G needle for both the 9:1 (*p* < 0.01) and 1:1 NO-OFP payloads. However, the difference for the NO-OFP-MB 1:1 v/v was not statistically significant (*p* = 0.12). For the NO-OFP-MB 9:1 v/v payload, the dose measured with the amperometric microelectrode sensor was 4.78 ± 1.03 mM and that derived from the size measurements was 5.57 ± 0.98 mM. The NO dose for the NO-OFP-MB 9:1 v/v payload measured with the sensor and the NO dose calculated from the size distribution measurements were not significantly different (*p* = 0.20). For the NO-OFP-MB 1:1 v/v payload, the dose measured with the sensor was 2.96 ± 0.61 mM and the dose derived from the size measurements was 2.02 ± 0.56 mM, which were also not significantly different (*p* = 0.20).

**Figure 10:**
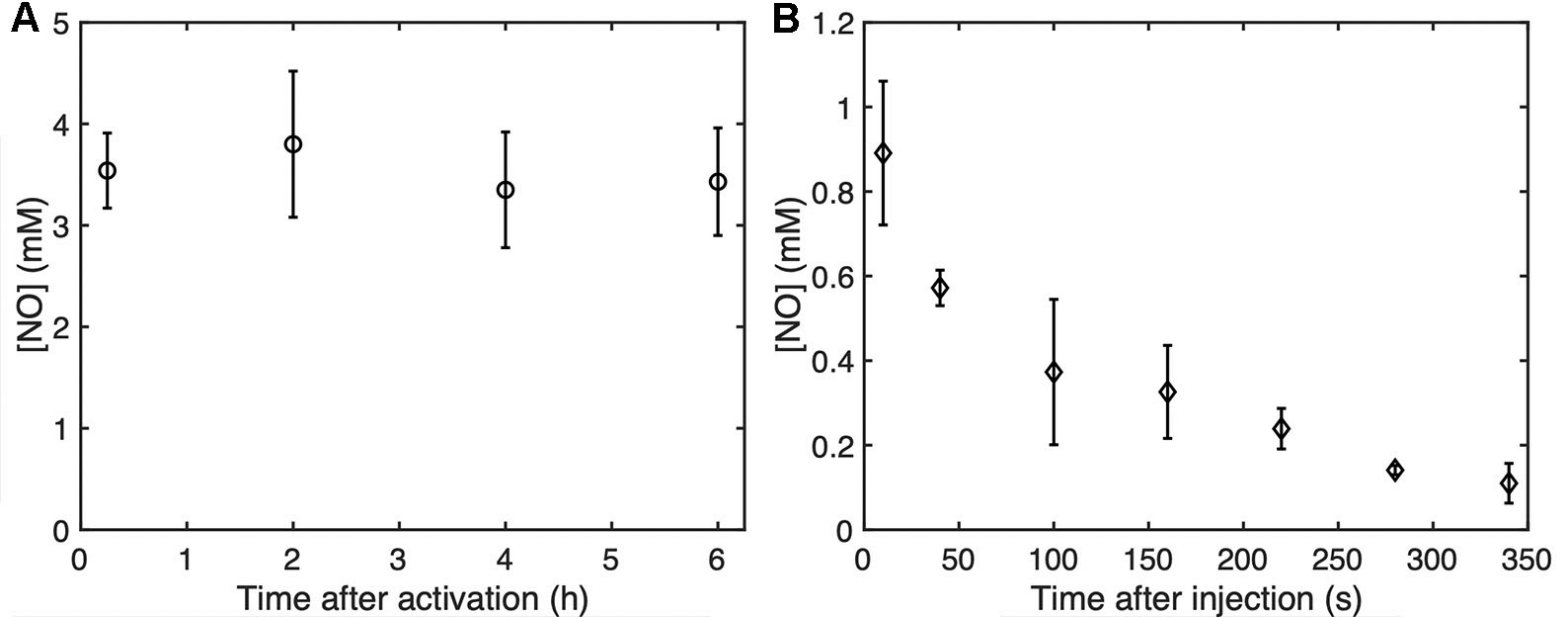
**(A)** The “in vial” concentration of NO-OFP-MB 9:1 v/v measured up to 6 hours after activation. **(B)** The NO concentration as a function of time up to 340 s after the addition of NO-OFP-MB 9:1 v/v in the 96 well-plate. The mean ± 1 standard deviation is depicted (n = 3 measurements for each time point). NO-OFP-MB, Nitric oxide and octafluoropropane microbubbles.

**Figure 10A** shows the NO dose in NO-OFP-MB measured at 15 min, 2, 4, or 6 h after activation. No change was observed in the dose over this period, which indicated “in vial” stability of the agent after activation at room temperature. **Figure 9B** shows the concentration of NO measured in air-saturated MH broth between 10 s and 340 s. A drop in the concentration of NO was observed over this period, with a half-life of approximately 50 s.

**Figure 11:**
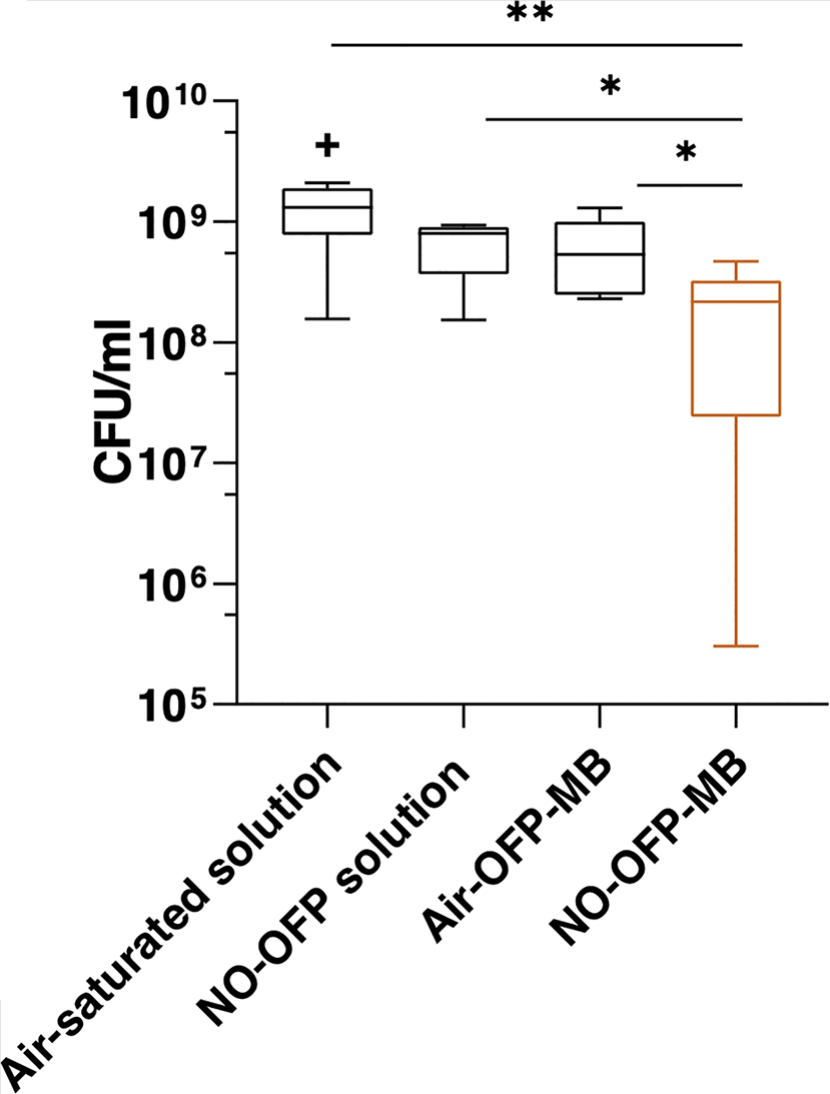
Measurement of bacteria colony forming units (CFU) after treatment with the solvent only (n = 10), NO-OFP-saturated solvent 9:1 v/v, (n = 9), air-OFP-MB 9:1 v/v, (n = 12), and NO-OFP-MB 9:1 v/v, (n = 12). The horizontal lines represent the median, the boxes represent the interquartile range, the whiskers represent 1.5-times the interquartile range. The symbols “*” and “**” represent *p*-values < 0.05, and < 0.01, respectively. The “+” sign represents a data point that lies outside the interquartile range by a factor > 1.5. NO-OFP-MB, Nitric oxide and octafluoropropane microbubbles.

**Figure 11** depicts the viability of *S. aureus* strain USA 300 after exposure to the MB solution, NO-saturated solution, air-OFP-MB 9:1 v/v, or NO-OFP-MB 9:1 v/v. NO-OFP-MB caused a statistically significant decrease in CFU of bacterial cells over air-OFP-MB (*p* < 0.05) and the NO-OFP-saturated solution (*p* < 0.05). There was no noticeable difference between the results of the experiments on three different days.

## Discussion

The frequency-dependent attenuation of NO-MB was substantially lower than NO-OFP-MB (**Figures 4** and **7**). Co-encapsulation of OFP, a perfluorocarbon gas was postulated to improve the stability of bioactive gas-loaded MB during the high-shear mixing process by allowing unshelled MB to persist until the formation of a lipid shell (Kwan et al., 2012). The osmotic and co-surfactant effect of OFP could be responsible for the enhancement of bubble formation and stability. The attenuation and total volume of NO-OFP-MB 9:1 v/v were similar to that of NO-OFP-MB 1:1 v/v, demonstrating that increasing the relative amount of OFP beyond 10% did not improve the gas loading. Nonetheless, it was demonstrated that the encapsulation of NO and OFP in MB enhanced bioactive gas loading relative to NO- and OFP-saturated solutions.

We have previously reported the ability to show ultrasound-responsive vasodilation in the presence of hemoglobin with NO-loaded cationic MB (or “bubble liposomes”) (Sutton et al., 2014). This agent had DPPC as the main lipid constituent. These cationic MB may help adhere to targets such as the vascular endothelium. The release of NO in close proximity to the target can help enhance delivery because NO is scavenged rapidly in the presence of hemoglobin or oxygen. However, cationic MB may also carry a higher risk for detrimental effects such as thrombosis.

In the present study, MB were prepared using DSPC (18-carbon long-chain lipid) as the primary constituent, which may enhance stability over DPPC (Borden and Longo, 2002). DIC images (**Figure 6**) reveal that our manufactured MB have a similar structure as Definity^®^ as previously reported (Shekhar et al., 2019a). A previous study employed the Griess assay in combination with serial dialysis for NO dose assessment within liposomes (Huang et al., 2009). This approach relies on colorimetric quantification of NO degradation products (nitrate and nitrite). This method is precise, but lacks temporal resolution and is based on indirect measurements. Our group has employed vasodilation of a viable artery *ex vivo* to assess the concentration of NO within MB (Sutton et al., 2014). This method has excellent temporal resolution, but lacks quantitative dose assessment. In the present study we used amperometric electrode sensing for direct measurements of NO concentration, which is highly specific to NO but has only moderate temporal resolution, approximately 5 s according to the manufacturer specifications. The dose measured with the NO sensor agreed with that calculated from the size measurement despite the high variability in both of these methods.

In this study, only a fraction of the NO payload was retained within the MB. Recent studies have shown the ability to stabilize bioactive gas payloads by co-encapsulating a small percentage of a perfluorocarbon gas (5%–20%) (Kwan et al., 2012; Yang et al., 2018). There are two main reasons why OFP does not diffuse across the lipid shell rapidly. The first reason is the very low solubility of OFP in water. This characteristic is expressed in Eq. 1 in the form of the Henry's constant. The solubility of OFP is 60-fold lower in water than oxygen and 200-fold lower than NO. This ratio proportionally impacts the quantity of gas escaping the bubble at each iteration of the simulation. Secondly, OFP is a bigger molecule than the other gases modelled. The collision diameter of OFP is twice that of NO or O_2_. As described in Eq. 3, the energy barrier created by the lipid layer induces a resistance to diffusion that depends on the exponential of the square of the collision diameter. The difference in collision diameters results in the lipid barrier retarding OFP diffusion by several orders of magnitude compared to smaller gases. However, the efficacy of perfluorocarbon gases for stabilizing payloads may depend on the physical properties of the bioactive gas. Perfluorocarbon gases with higher molecular weights than OFP could also be explored for stabilizing NO payloads.

The lipid-shelled agents used in the present study were pegylated. PEG brushes serve as emulsifying agents, preventing MB aggregation (Chen and Borden, 2010). The presence of PEG has been reported to cause a loss of viability in bacterial culture (Chirife et al., 1983; Nalawade et al., 2015), which could explain the drop in CFU/ml in the air-OFP-MB group relative to the air-saturated solution (**Figure 11**). This decrease was not statistically significant (p = 0.15). We hypothesize that the small amount of PEG used in the present formulation was likely not sufficient to have a demonstrable antibacterial effect. Nonetheless, NO-OFP-MB showed a statistically significant enhancement in bactericidal activity beyond both air-OFP-MB and NO-OFP-saturated solution. The variability in the efficacy could be attributed to variability of the fluid mixing inherent to the manual syringe injection of the solution into the well plates, and the variability in NO-loading of the MB (see **Figure 9**). Although the decrease in CFU/ml was modest, the present study provides a proof-of-principle for using NO encapsulated in a lipid-shelled agent for bactericidal applications.

NO is a highly diffusive gas, which makes it challenging to prevent passive release of the gas through a microbubble shell an over extended timescale. In this study, the temporal response of the amperometric microelectrode sensor combined with the time taken to stir the solution was slow (~5–7 s). Therefore, the passive release profile of NO from the agent could not be determined precisely. In addition, the interaction of NO with dissolved oxygen and the rapid degradation of NO made the assessment of the passive release profile challenging. MB stabilized with other types of shell materials, such as polymers (McEwan et al., 2014), dextran (Cavalli et al., 2009a), surfactants (Eisenbrey et al., 2015; Eisenbrey et al., 2018), or chitosan (Cavalli et al., 2009b) shells, may increase the timescale of gas diffusion across the shell, with or without the addition of osmotic gases for payload stabilization. The efficacy and biocompatibility of such agents for encapsulating NO could also be compared to lipid-shelled MB.

In the present study, both the NO dose assessment and bactericidal experiments were performed in an aerobic environment because the antibacterial effect of NO has been reported to require an aerobic environment to form reactive nitrosative and oxidative intermediates (Fang, 1997). However, NO also degrades rapidly following second-order kinetics in the presence of dissolved oxygen to form nitrate and nitrite (Howlin et al., 2017). Therefore, oxygen plays a paradoxical role in NO-mediated bactericidal applications.

NO-OFP-MB were stable inside the vial for at least 6 h after activation (**Figure 10**). After formation of the MB, the vial solution (mixture of PBS, propylene glycol and glycerol) likely became saturated with NO. The NO-saturated environment could have reduced the diffusion gradient and kept the MB stable. If dilution of NO-OFP-MB is required for antibacterial applications, the agent may be diluted in NO and OFP-saturated solutions to keep the agent stable before ultrasound-mediated release. Due to the presence of oxygen in air-saturated media, the concentration of NO in the plate wells decreased rapidly over 340 s. A similar result was obtained by calculating the NO diffusion and scavenging by oxygen in water. The measured NO concentration was lower, which could be due to additional mixing caused by pipetting while transferring the sample from the 96 well-plate to the NO measurement chamber. The bacteria were likely exposed to NO only for a 5–6 min, which could explain why only a modest enhancement in bacterial killing was observed in this study. Note that previous studies have relied on prolonged exposure to NO, albeit at lower concentrations for antibacterial therapy (Barraud et al., 2009; Schairer et al., 2012; Barraud et al., 2014; Howlin et al., 2017).

This study evaluated the antibacterial efficacy on a single *S. aureus* strain, USA 300, which is one of the most common community-associated Methicillin-resistance (MRSA). This organism causes skin and soft issue infections, along with more invasive conditions such as bacteremia, endocarditis, osteomyelitis, and severe necrotizing pneumonia (Tenover and Goering, 2009). Future studies will be needed to evaluate the effect of NO-OFP-MB on other common bacterial strains. Furthermore, bactericidal effects of NO-OFP-MB were evaluated in a planktonic culture. Further research should be conducted to assess this strategy on the dispersal and killing of biofilms. Although we demonstrated a statistically significant enhancement in bacterial killing with respect to air-OFP-MB and NO-OFP-saturated solution, the effect of this therapy on host cells also needs to be evaluated to assess off-target effects. Studies using *in vivo* models should be performed to assess the therapeutic efficacy of NO-OFP-MB. These agents could also be combined with antibiotics (Ren et al., 2016) and other antimicrobial agents (Privett et al., 2010; Worley et al., 2014; Storm et al., 2015) to enhance therapeutic efficacy.

The reduction in ultrasound-triggered attenuation showed the feasibility of releasing the bioactive gas payload by destroying the lipid-shelled MB (**Figure 8**). The rapid release of NO from NO-OFP-MB for over either milliseconds (using ultrasound) or several seconds (passively) at the target site could avoid unwanted systemic effects, such as hypotension, which are a limitation of chemical NO donors. The rapid passive release of NO from lipid-shelled MB is a “double-edged sword” as it dictates that these agents should be delivered in the vicinity of the target using either direct injection or a catheter, but limits potential off-target effects. NO-OFP-MB may be useful for various applications, such as treating infections related to dialysis vascular access (Saeed Abdulrahman et al., 2002), and for infections in the urinary tract (Pietropaolo et al., 2018), in which the target tissue may be easily accessible. The formulation could be delivered directly to the target site using a catheter, and the gas payload released either rapidly using ultrasound, or passively by diffusion. Either a bolus or a diluted bolus in NO-saturated saline could be employed to retard loss of gas due to diffusion before reaching the target. Indwelling catheters could also be treated using this route of administration. NO-OFP-MB could also be used to treat biofilms on infected skin wounds with topical administration along with ultrasound exposure at the site of interest.

In summary, we demonstrated that NO can be loaded into lipid-shelled MB with OFP gas that increased the NO dose relative to NO- and OFP-saturated solutions. The attenuation and total volume of NO-OFP-MB was higher than lipid-shelled bubbles fabricated with NO alone. The feasibility of antibacterial therapy with NO-OFP-MB against a resistant USA 300 strain was demonstrated. A statistically significant enhancement in bacterial killing over NO-OFP-saturated solution and air-OFP-MB was demonstrated *in vitro* with NO-OFP-MB. These results suggest that lipid-shelled MB loaded with NO and OFP could be useful for bactericidal applications.

## Data Availability Statement

The datasets generated for this study are available on request to the corresponding author.

## Author Contributions

HS, ML, WP, AP, SC, and CM performed experiments and data analysis. ML performed the numerical modeling with CH guidance. CH and DH guided the experimental design. HS and ML wrote the first draft of the manuscript, which was critically revised and approved by all authors.

## Funding

This work was funded by the U.S. National Institutes of Health, National Institute of Neurological Diseases and Stroke through grant R01 NS047603. DH was supported by ARCH Biopartners, Inc. (Toronto, CA). The funder was not involved in the study design, collection, analysis, interpretation of data, the writing of this article or the decision to submit it for publication.

## Conflict of Interest

CH, HS, and AP are inventors on a patent application #15788224 entitled “Gas-Encapsulated Acoustically Responsive Stabilized Microbubbles and Methods for Treating Cardiovascular Disease” published on 8th February 2018.

The remaining authors declare that the research was conducted in the absence of any commercial or financial relationships that could be construed as a potential conflict of interest.
